# Partial-Thickness Burns of the Posterior Trunk Treated With Combined Autologous Skin Cell Suspension and Meek Grafting

**DOI:** 10.7759/cureus.106821

**Published:** 2026-04-10

**Authors:** Junya Oshima, Yoshiaki Inoue, Kaoru Sasaki, Mitsuru Sekido

**Affiliations:** 1 Department of Plastic, Reconstructive, and Hand Surgery, University of Tsukuba, Tsukuba, JPN; 2 Department of Emergency and Critical Care Medicine, University of Tsukuba, Tsukuba, JPN

**Keywords:** autologous skin cell suspension, donor site conservation, meek grafting, partial-thickness burns, posterior trunk burns

## Abstract

Burns of the posterior trunk are challenging to manage due to constant pressure, shear stress, and exudate accumulation, which increase the risk of graft detachment and infection. Autologous skin cell suspension (ASCS) is effective for donor-site conservation but may be difficult to retain on posterior trunk wounds. We report two cases of partial-thickness burns treated with ASCS in combination with high-expansion (1:9) Meek grafting. In both cases, debridement was performed using a hydrosurgical system, followed by the application of ASCS and Meek grafts. Rapid epithelialization was achieved, and donor skin harvesting was minimized. These results suggest that combining ASCS with Meek grafting may facilitate wound healing and donor-site conservation in posterior trunk burns, which are often difficult to manage.

## Introduction

Burns of the posterior trunk are common injuries; however, wound and graft management in this region are often challenging. This is due to constant pressure and shear stress, as well as the gravitational accumulation of exudate, which can create a relatively enclosed environment and increase the risk of infection.

Autologous skin cell suspension (ASCS) monotherapy is an effective treatment option for partial-thickness burns and is particularly useful for donor-site conservation. However, because ASCS is applied as a liquid to the wound surface, cell retention may be difficult in posterior trunk wounds; therefore, ASCS monotherapy is often avoided in this region.

Here, we report two cases in which favorable results were achieved using ASCS in combination with high-expansion Meek grafting for partial-thickness burns of the posterior trunk.

## Case presentation

Case 1

**Figure 1:**
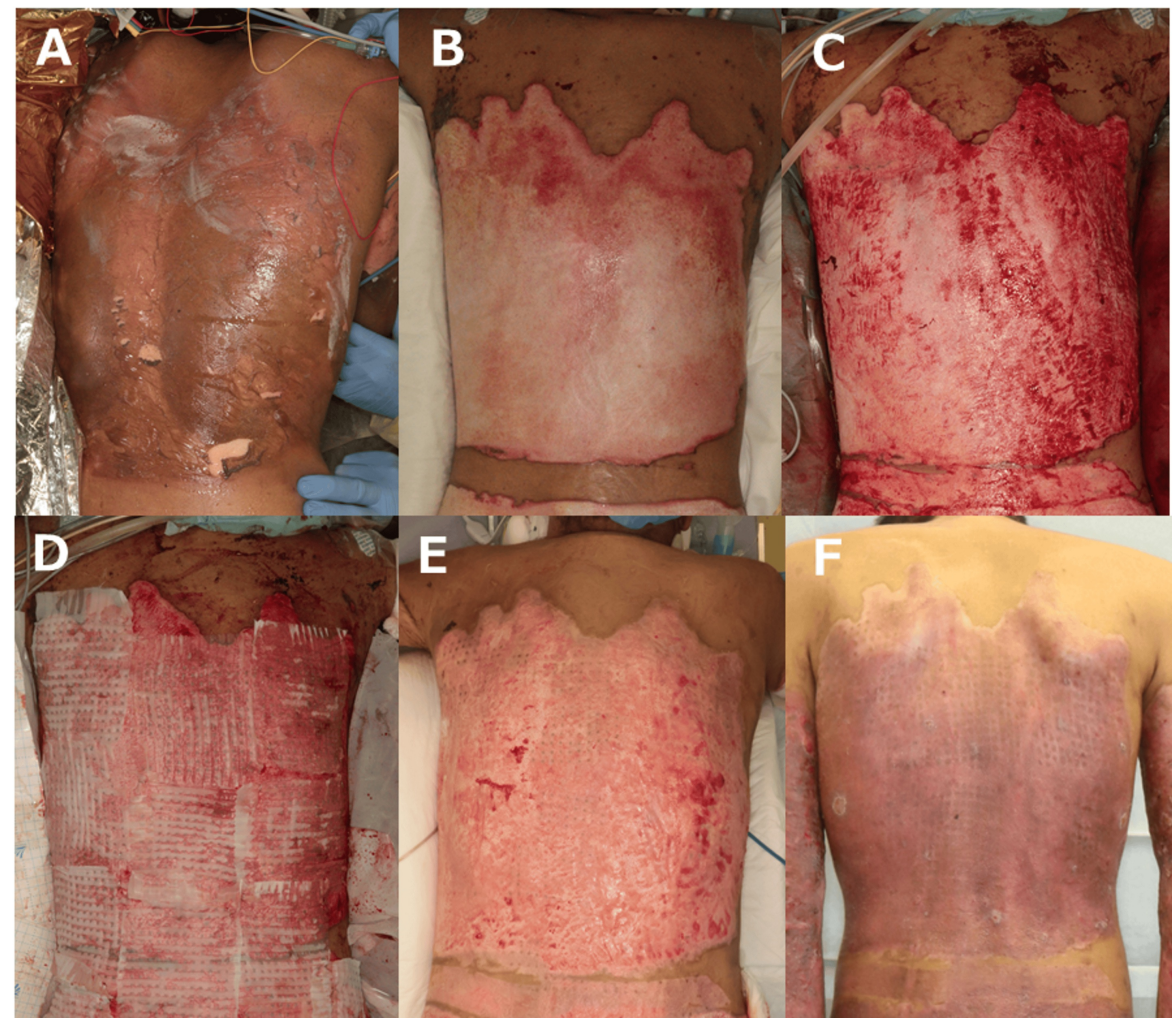
Clinical course of Case 1. (A) Findings on admission: burn wounds involving the entire back. (B) Intraoperative findings before debridement, showing yellow necrotic tissue. (C) Findings after debridement. (D) Findings immediately after the application of ASCS and Meek grafts. (E) Postoperative day 14: epithelialization was largely achieved. (F) Six weeks after surgery: no significant scar thickening or inflammation was observed. ASCS: Autologous skin cell suspension.

A 26-year-old man sustained burn injuries when his clothes caught fire while cooking. He had burns involving 51% of the total body surface area, including the back, groin, thighs, and both upper limbs (Figure 1A). On day 12 post-injury, debridement was performed using a hydrosurgical system (Figure 1B-1C). On the same day, 1:9 Meek skin grafts and ASCS were applied (Figure 1D). ASCS was prepared using a commercially available kit (Avita Medical). On postoperative day 8, the contact layer was removed, and survival of the Meek grafts was confirmed. By postoperative day 14, although a small area of unepithelialized wound remained, overall epithelialization had been achieved (Figure 1E). The patient was discharged six weeks after surgery (Figure 1F).

Case 2

**Figure 2:**
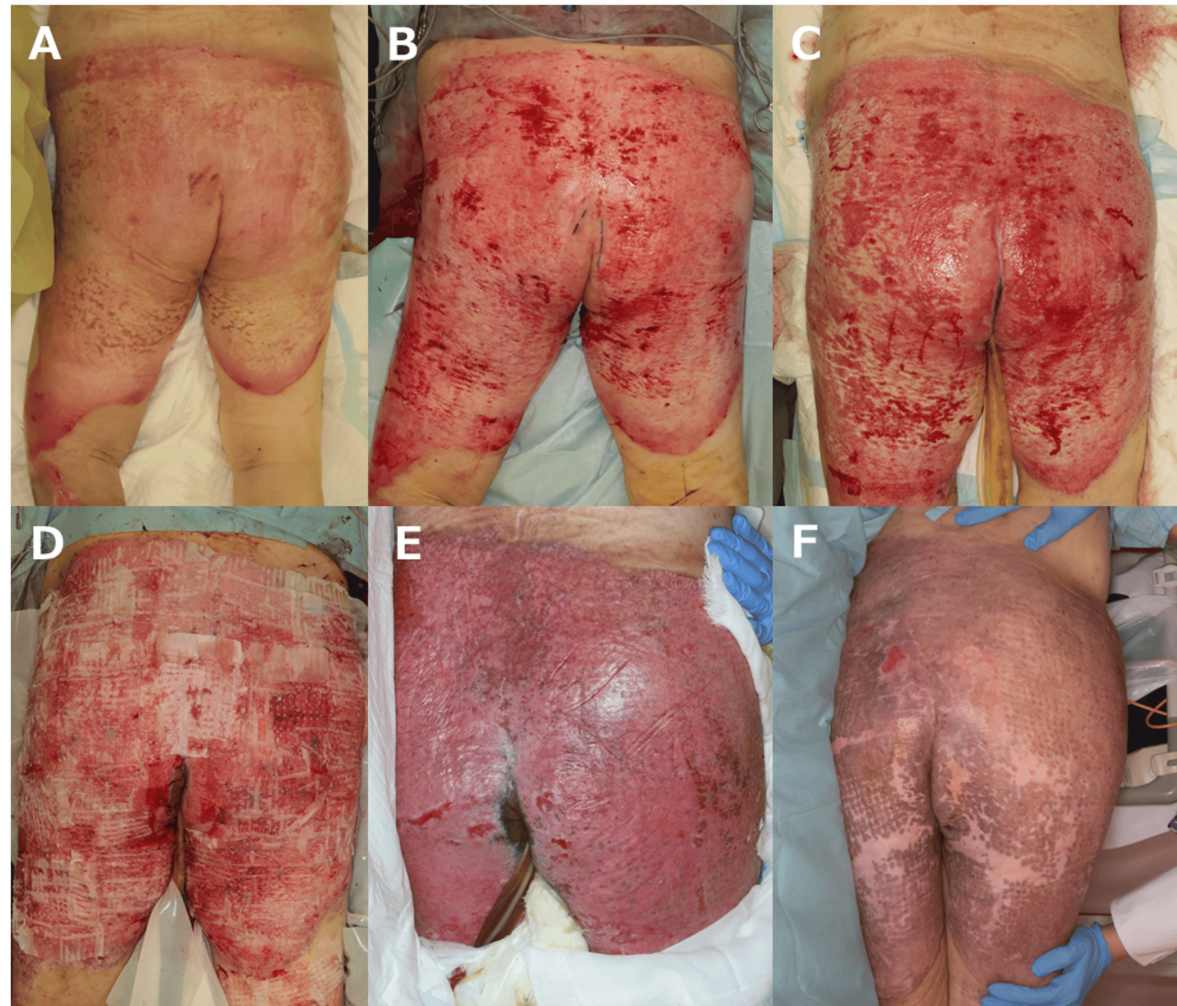
Clinical course of Case 2. (A) Intraoperative findings during the first surgery before debridement, showing partial-thickness burns predominantly involving the buttocks. (B) Findings after debridement. The initial procedure involved the application of ASCS and dressings. (C) Sixteen days after ASCS application: adequate epithelialization was not observed, and reoperation was planned. (D) Findings during the second surgery, immediately after the application of ASCS and Meek grafts. (E) Thirteen days after grafting: epithelialization was largely achieved. (F) Eight weeks after surgery: no significant thickening or inflammation was observed. ASCS: Autologous skin cell suspension.

A 79-year-old woman sustained burn injuries after becoming immobile in a bathtub filled with hot water. She had burns involving 27% of the total body surface area, including the buttocks, posterior thighs, soles of the feet, and lower abdomen (Figure 2A). The first surgery was performed on day 8 post-injury. Debridement was performed using a hydrosurgical system, and ASCS monotherapy was applied on the same day (Figure 2B). However, adequate epithelialization was not achieved (Figure 2C), and a second surgery was performed on day 24 post-injury. After refreshing the wound surface with a hydrosurgical system, 1:9 Meek skin grafts and ASCS were applied (Figure 2D). On postoperative day 8, the contact layer was removed, and survival of the Meek grafts was confirmed. Epithelialization was achieved 13 days after grafting. The patient was discharged eight weeks after surgery.

## Discussion

Burns of the posterior trunk are common; however, postoperative management is often challenging. Because patients spend most of their time in the supine position, this region is continuously subjected to pressure and shear stress, which can compromise graft survival. In addition, frequent repositioning may increase the risk of graft displacement. Another challenge is the accumulation of wound exudate in the posterior trunk. Hematoma and serous fluid collection can contribute to graft detachment. Furthermore, in the supine position, this region may function as a relatively enclosed space, making frequent observation more difficult than in other areas. Under these conditions, the accumulation of exudate may increase the risk of infection. Air-fluidized beds are useful for managing posterior trunk burns [1]; however, their high cost and maintenance complexity limit their availability in many facilities. Although prone positioning is an alternative, it presents challenges related to respiratory management, increased abdominal pressure, and the risk of anterior pressure ulcers [2].

In the management of posterior trunk burns, burn depth is an important factor in determining the treatment plan [3]. Full-thickness burns generally require surgical intervention with skin grafting, whereas superficial partial-thickness burns are typically managed conservatively. However, the optimal approach for deep partial-thickness burns, including deep dermal burns, remains controversial. Given the challenges in postoperative management described above, conservative treatment may be preferable in some cases. However, conservative treatment is associated with prolonged healing times and may increase the burden of wound care and the risk of infection. Therefore, early wound closure through surgical intervention may be a more appropriate strategy in selected cases.

ASCS is a regenerative medicine technique in which a small sample of the patient’s skin is enzymatically processed to produce a suspension containing keratinocytes, melanocytes, fibroblasts, and other cells, which is then applied to the wound surface [4]. A key advantage of this technique is that a small donor skin sample can be used to treat a burn area up to approximately 80 times its original size. In full-thickness burns, ASCS used in combination with conventional skin grafting may reduce the amount of donor skin required and promote epithelialization. In partial-thickness burns, ASCS monotherapy may be sufficient, allowing treatment with minimal donor skin harvesting [5].

ASCS monotherapy may be an effective option for partial-thickness burns of the posterior trunk; however, its clinical application can be limited by poor cell retention. Because ASCS is applied as a liquid, it may be difficult to maintain adequate contact with the wound surface in the supine position. In Case 2, initial treatment with ASCS monotherapy resulted in insufficient cell retention and inadequate epithelialization. Therefore, high-expansion Meek grafting was used as a carrier for ASCS. Meek grafting is a technique that enables high-expansion skin grafting by dividing the skin into small, uniformly distributed islands [6]. Meek grafts offer several advantages. Because each graft island is independent, partial graft loss is less likely to spread to adjacent areas. Furthermore, the small graft size is associated with lower metabolic demand, which may contribute to high engraftment rates and resistance to infection [7,8].

In this study, highly expanded Meek grafts were used as carriers for ASCS. The combined use of Meek grafting may facilitate retention of the cell suspension on the wound surface, thereby promoting cell proliferation and rapid epithelialization. Reducing the Meek expansion ratio may diminish the donor-sparing advantage of ASCS; therefore, a 1:9 expansion ratio was selected. Although Meek grafting alone is effective for posterior trunk burns, epithelialization with 1:9 Meek grafting alone has been reported to require approximately one month after surgery [9]. In our cases, epithelialization between graft islands was observed at approximately two weeks, suggesting that this combined approach may accelerate wound closure in posterior trunk burns, which are often difficult to manage. A limitation of this method is that it requires a greater amount of donor skin than ASCS monotherapy. However, epithelialization may be achieved using donor skin corresponding to approximately one-eighth of the burn area (1/80 + 1/9 ≈ 0.123 ≈ 1/8). Therefore, this technique represents a useful option for the management of challenging posterior trunk burns.

## Conclusions

The use of high-expansion Meek grafts as carriers for ASCS may facilitate rapid epithelialization and reduce donor-skin requirements in posterior trunk burns. This combined approach may represent a practical strategy for managing these challenging wounds.
